# A randomized controlled trial on the comparative effectiveness of mindfulness-based cognitive therapy and health qigong-based cognitive therapy among Chinese people with depression and anxiety disorders

**DOI:** 10.1186/s12888-020-02994-2

**Published:** 2020-12-14

**Authors:** Sunny H. W. Chan, Wendy W. K. Chan, June Y. W. Chao, Phyllis K. L. Chan

**Affiliations:** 1 Department of Rehabilitation Sciences, The Hong Kong Polytechnic University, Hong Kong, China; 2 Department of Occupational Therapy, Queen Mary Hospital, Hong Kong, China; 3 Department of Psychiatry, Queen Mary Hospital, Hong Kong, China

**Keywords:** Mindfulness, Qigong, Cognitive behavior therapy, Mood disorders, Chinese culture

## Abstract

**Background:**

The goal of this study was to investigate treatment outcome and related intervention processes of mindfulness-based cognitive therapy versus health qigong-based cognitive therapy versus waitlist control among individuals with mood disorders.

**Methods:**

A total of 187 individuals with mood disorders were randomized and allocated into mindfulness-based cognitive therapy, health qigong-based cognitive therapy, or waitlist control groups. All participants were assessed at three time points with regard to depressive and anxiety symptoms, physical and mental health status, perceived stress, sleep quality, and self-efficacy. Linear mixed models analysis was used to test the individual growth model by studying the longitudinal data.

**Results:**

Mindfulness-based cognitive therapy and health qigong-based cognitive therapy both produced greater improvements on all outcome measures as compared with waitlist control. Relatively, more reductions of mood symptoms were observed in the health qigong-based cognitive therapy group as compared with the mindfulness-based cognitive therapy group. Health qigong-based cognitive therapy is more conducive to physical health status whereas mindfulness-based cognitive therapy has more favorable mental health outcomes. Individual growth curve models indicated that alterations in perceived stress was the common predictor of mood changes in both intervention groups.

**Conclusions:**

The predominant emphasis on physical health in health qigong-based cognitive therapy makes it more acceptable and effective than mindfulness-based cognitive therapy as applied in Chinese individuals with mood disorders. The influence of Chinese culture is discussed.

**Trial registration:**

HKU Clinical Trials Registry. Identifier: HKUCTR-2558. Registered 21st Nov 2018.

**Supplementary Information:**

The online version contains supplementary material available at 10.1186/s12888-020-02994-2.

## Background

Mood disorders such as depression and anxiety are very prevalent in China [1], with both of them are among the top 10 causes of disease burden worldwide [2]. Thus, it has prompted the necessity to find ways for better treatment and planning of care. Individuals may consider psychotherapy instead of pharmaceutical treatment such as selective serotonin reuptake inhibitors or benzodiazepine due to possible side effects (e.g., headache) and potential dependence on medication, respectively [3–5]. Cognitive behavioral therapy (CBT) has been substantially proven to be an effective psychosocial treatment in managing depression and anxiety [6, 7]. However, some reviews showed that the effect sizes of CBT for depression have steadily decreased since its inception four decades ago [8–10]. Thus, merely employing CBT might not be sufficient in managing mood disorders.

In view of the limitations of mainstream treatments, much attention shifted to alternative forms of therapy. There is a growing interest in studying mind-body interventions (MBIs) for treating depression and anxiety [11, 12]. Mindfulness meditation (MM) is one of the prominent examples of MBIs. Review research and meta-analyses have demonstrated its effectiveness in treating depression and anxiety [13, 14]. An integration of MM with CBT was suggested to be an effective approach for psychological health and mood symptoms [15, 16]. However, MM should only represent the static form of MBIs. Traditionally, there is another dynamic form of mind-body interventions with a focus on body movement, which is called health qigong (HQ [17];). HQ can be viewed as a kind of physical movement with introspective focus of breathing and energy in the body [18]. HQ should be able to reduce depression or anxiety in people with physical or mental illness [19, 20]. According to a speculative review, the combination of HQ with CBT was proposed as one of the major behavioral strategies in countering mood symptoms [21]. There was an attempt to apply the combined form on elderly people [22], but the application on people with mood disorders is still waiting for further investigation.

Taken together, MM and HQ are both perceived as MBIs and share the common features of a focus on breathing, but they represent two distinct approaches with the emphasis on mind-based practice and body-based movement practice, respectively. The integration of the former with CBT has been proven to be effective in improving mood symptoms. However, the integration of HQ and CBT still requires further research. Moreover, the above-reviewed studies of either MM or HQ seldom used a comparable mind-body intervention for control which may limit the utility in informing clinical practice. In addition, originating as a traditional Chinese health and fitness exercise [17], HQ appears to be a preferred form of non-pharmacological therapy for Chinese adults [23]. Thus, it is worthwhile to fill the research gaps by comparing two different forms of MBIs, when integrated with CBT, in a Chinese population. Furthermore, studies suggested that MM is more conducive to mental health condition [24, 25] whereas HQ would be more effective in treating physical health outcome [26, 27]. Besides, research also demonstrated that both MM and HQ showed certain effects on stress reduction, improvement of sleep quality, and enhancement of self-efficacy [17, 25, 28, 29]. The evidence of alternative health outcomes brought about by combining CBT with these two different forms of MBIs is thus worthy of further investigation. Further investigation is warranted to fill these research gaps.

The overarching objective of the present study was to assess and compare the relative therapeutic effects of mindfulness-based cognitive therapy (MBCT) and health qigong-based cognitive therapy (HQCT) in treating depression and anxiety with a waitlist control (WC) group in a Chinese context. There are three hypotheses in this study: (1) MBCT and HQCT would lead to improvements in primary outcome (mood symptoms) as well as secondary outcomes (physical and mental health statuses, perceived stress, sleep quality, and self-efficacy) as compared with the WC group; (2) MBCT should be more favorable to enhancing mental health outcomes than HQCT; whereas HQCT is better in improving physical health outcomes than MBCT; (3) Participants in the HQCT group would have a greater improvement in mood symptoms than counterparts in the MBCT group in a Chinese context.

## Method

### Participants

The present study adopted a three-arm randomized controlled trial (RCT) with waitlist control design. Participants were recruited from a psychiatric outpatient clinic in Hong Kong. Inclusion criteria included: (i) a diagnosis of depression or anxiety disorder based on Diagnostic and Statistical Manual of Mental Disorders (DSM-IV [30];) (the diagnoses were confirmed by attending psychiatrists); (ii) age between 18 and 70 years; (iii) regular psychiatric follow-up; (iv) no suicidal tendencies; (v) primary education level or above; (vi) no previous experience with cognitive therapy, mindfulness-based intervention, or health qigong. Individuals with comorbid diagnoses of schizophrenia, schizoaffective disorder, substance misuse, organic brain syndrome, personality disorder, or intellectual disabilities were excluded.

**Fig. 1:**
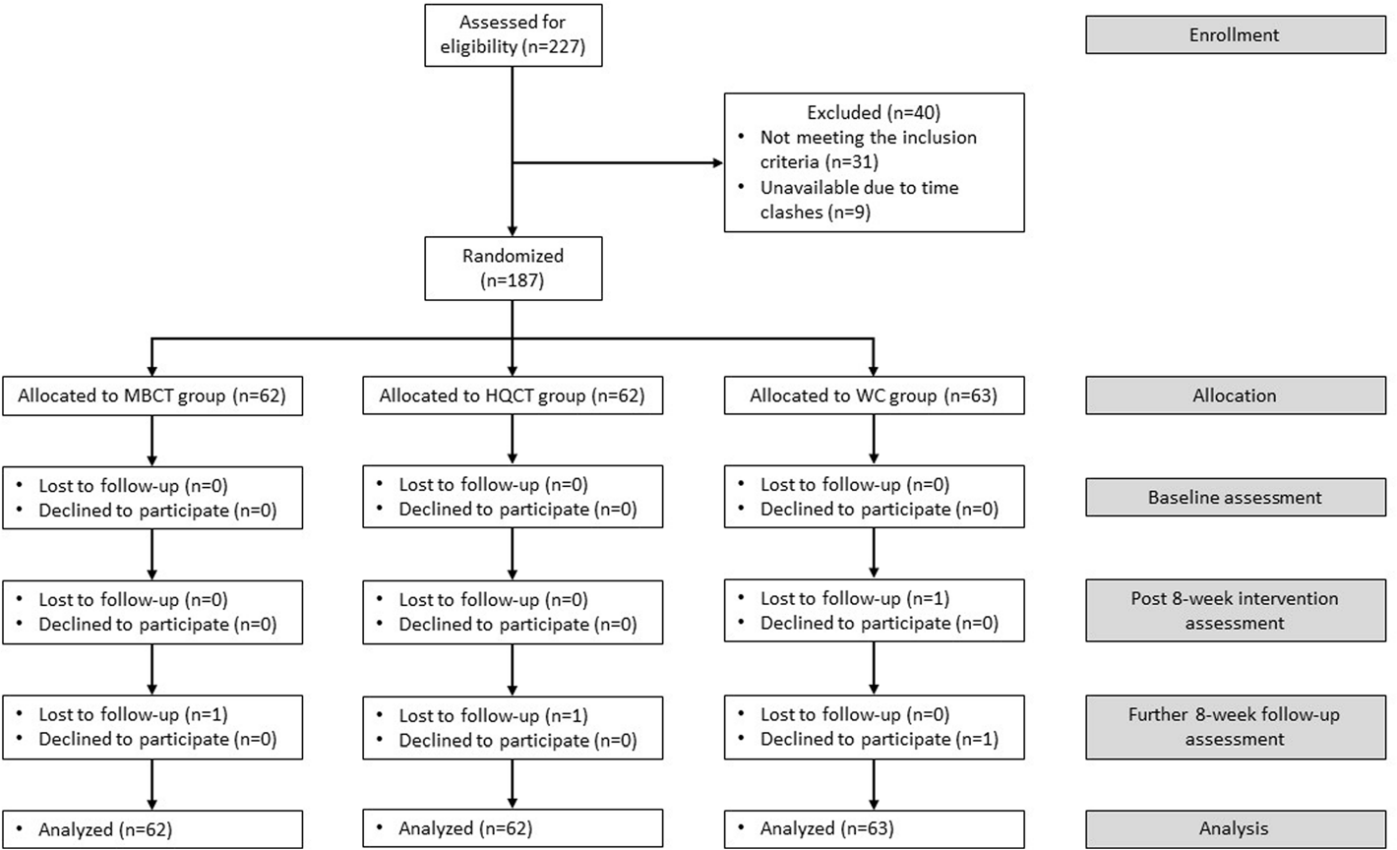
Consort flow diagram of participants through the study

According to the G*Power programme [31], in order to achieve a statistical power of 0.8 with a small effect size (f = 0.2) and a significance level of 0.05 in repeated measures, a multivariate analysis of variance under the proposed three-group, three-time-point design and a total sample size of 152 will be needed. With an estimated attrition rate of about 15%, a final sample size of 180 will be needed. A total of 227 individuals showed interest to join the research. They were assessed for eligibility and 40 of them were excluded as they did not meet the inclusion criteria or were unavailable in the scheduled time. Finally, informed consent was obtained from 187 eligible and available participants which are more than the minimum requirement. After the initial baseline assessment, participants were randomly assigned to one of the study arms using a list of computer-generated random numbers. The generation of random numbers and their assignment was performed by a statistician who is unaware of the research project’s aims. Another research assistant, who assisted with the outcome assessment and data analysis, was kept unaware of the group allocation results. Through simple randomization, participants were assigned to one of the study arms in a ratio 1:1:1, namely, (a) MBCT; (b) HQCT; and (c) WC group. The WC group would continue with routine care and could join either MBCT or HQCT after 16 weeks (post final assessment). Participants in both intervention groups were monitored of not taking part in other psychosocial interventions along the whole research period. Any adverse event was also observed throughout the whole process of intervention. During the entire research follow-up, a total of four participants dropped out of the study (2.14% attrition rate). The flow chart of the study is depicted in Fig. 1. Ethical approval was obtained from the Institutional Review Board of the University of Hong Kong/Hong Kong West Cluster of the Hospital Authority (UW18–458). The study adheres to CONSORT guidelines.

### Interventions

MBCT is a manualised eight-week group program incorporating various mindfulness and cognitive restructuring techniques [32]. The intervention consists of eight weekly sessions of about 2 hours each in terms of formal and informal practice during which participants are guided to use breathing as an anchor for sustained attention in the present moment. The whole program incorporates a body scan technique, sitting meditation, mindful walking, mindful hatha yoga, mindful movement, and other mindfulness activities linked to ordinary daily activities. The details of each session and the entire program was illustrated thoroughly in the major MBCT manual [32]. Qualified therapists with basic professional training in mindfulness-based interventions, in addition to at least 2 years’ experience conducting mindfulness-based programs, were invited to implement the MBCT group. HQCT is a program of eight weekly sessions with a combination of teaching CBT and HQ techniques. CBT materials were adapted from a Changeways core program [33] which is a psychoeducational group therapy protocol designed for people with mood disorders. The protocol covers a variety of problem-solving and lifestyle management skills such as identifying negative thought patterns and cultivating adaptive and positive thinking. Baduanjin was chosen as the HQ practice because it has been perceived as less complicated and without restrictions on time and space [34, 35]. A complete cycle of Baduanjin took 10–15 min for completion, which consisted of eight sequential and simple forms of movements. A detailed description of the physical movements in Baduanjin protocol was reported elsewhere [17]. In addition, Baduanjin is also perceived as the major behavioral strategy when delivering CBT. The entire intervention also consists of eight weekly sessions of about 2 hours each. Qualified therapists with basic professional training in CBT and HQ were invited to implement the HQCT group. Participants in the WC group received treatment as usual without any additional intervention.

### Measures

Assessments took place at three time points according to the following time schedule: (i) baseline (T0); (ii) post intervention after 8 weeks (T1); (iii) follow-up after a further 8 weeks (T2). All assessments were validated in the Hong Kong Chinese population and were conducted by trained researchers. The primary outcome measure included the Chinese version of the 21-item Depression Anxiety and Stress Scale (DASS-21 [36, 37];) which is a measure of mood symptoms. The secondary outcome measures included physical and mental health statuses as assessed by the Chinese (HK) Short-form-12 (SF-12 [38];); perceived stress as evaluated by the Chinese Perceived Stress Scale (CPSS [39];); sleep quality as assessed by the Chinese version of the Pittsburgh Sleep Quality Index (PSQI [40];); and general self-efficacy as tested by the Chinese General Self-efficacy Scale (CGSS [41];). Other socio-demographic data including age, gender, and educational level were obtained from medical records.

### Data analysis

We analyzed the data using SPSS version 24.0. An ANOVA was conducted for the continuous variables and a chi-square test was used for the categorical variables, to compare the differences between the primary and secondary outcomes of the three groups at baseline. We used the intention-to-treat principle and linear mixed models (LMM) with restricted maximum likelihood estimation. Both intra- and interindividual differences in the growth parameters, including intercepts and slopes, were further investigated through individual growth curve (IGC) models [42]. IGC can also explore the causal links between the time variant or time invariant predictors and changes in outcome variables across time. LMM usually create a two-level hierarchical model that nests time within individual: Level 1, or the within-person or intraindividual change model, is the model for repeated measures with time variant variables nested within individuals, whereas Level 2, or the between-subject model, is the model for time invariant variables between groups of individuals.

We started the analysis with the unconditional mean model that did not contain any predictors, and then we fitted the linear and the quadratic growth models to see the change in outcome measures over time. At Level 1, we used the autoregressive covariance structure (AR1) with homogeneous variances [43, 44]. At Level 2, we chose a better model based on a smaller value of the Akaike information criterion (AIC). In addition, for comparison of group differences, we also reported effect sizes as Cohen’s d [45]. Finally, individual growth model with time invariant and time variant predictors was conducted for both intervention groups.

## Results

### Demographic characteristics and clinical outcome variables

**Fig. 2:**
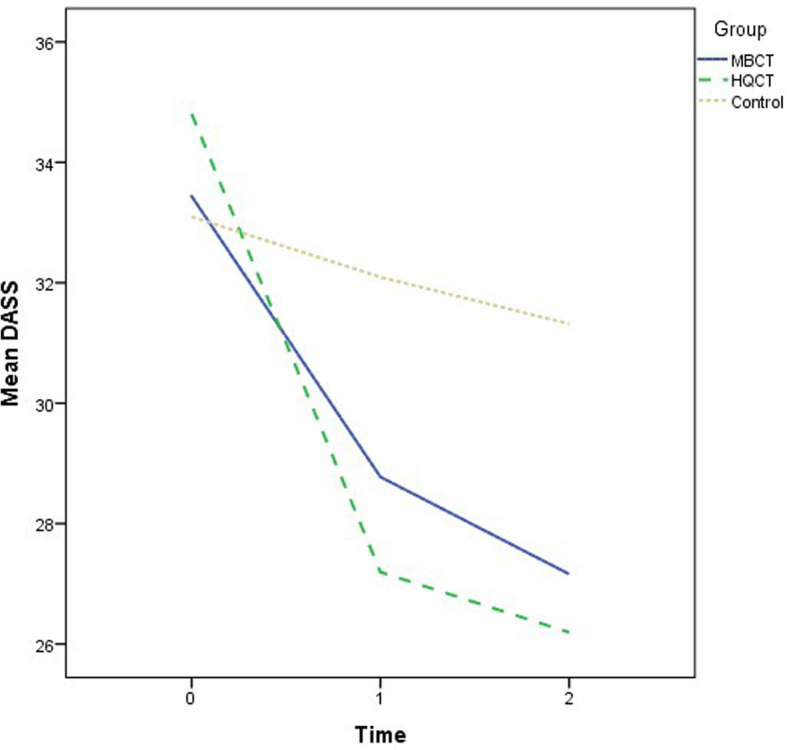
Growth trajectories of mood symptoms for the three groups across three time points

**Fig. 3:**
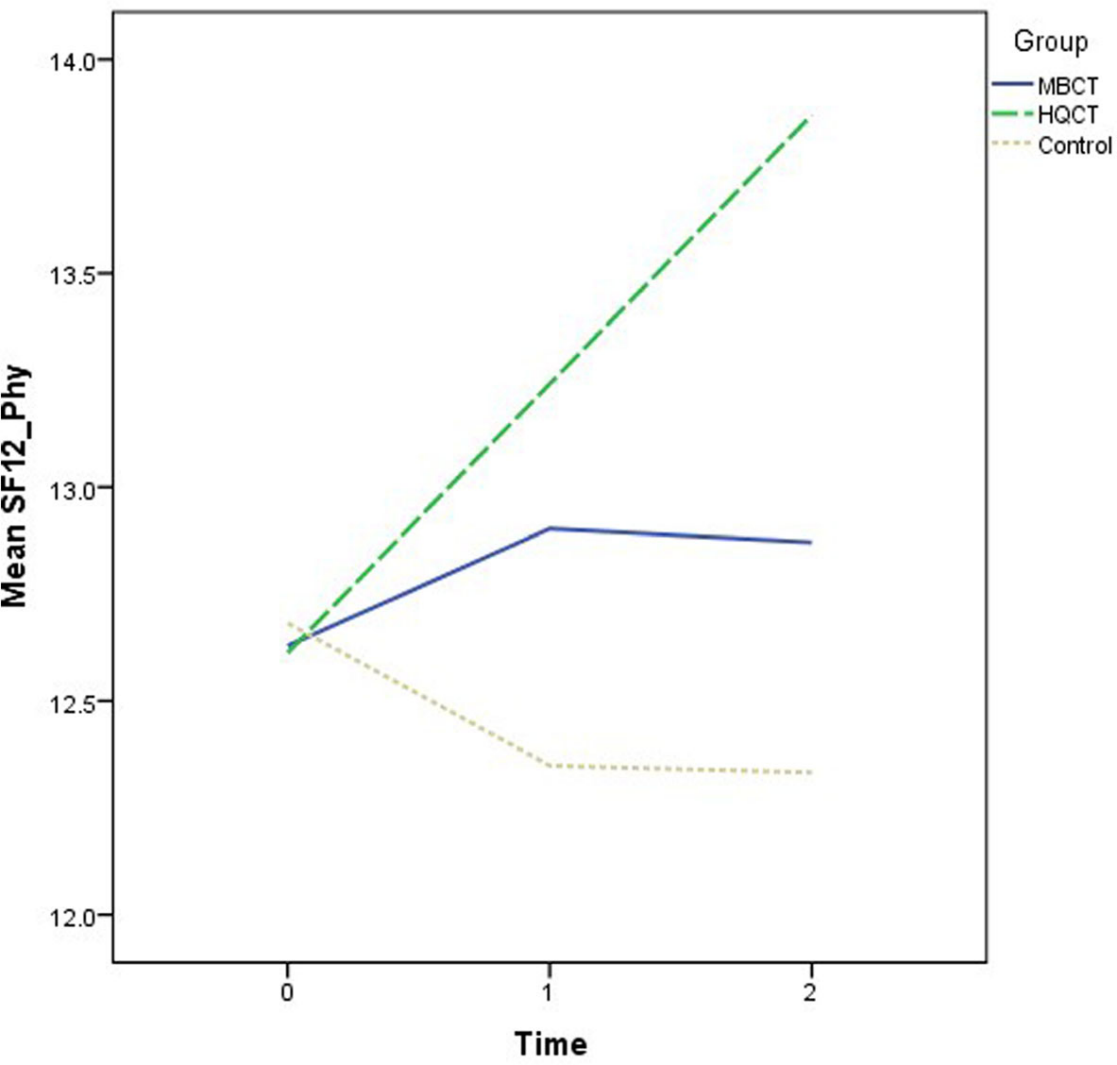
Growth trajectories of health status (Physical) for the three groups across three time points

**Fig. 4:**
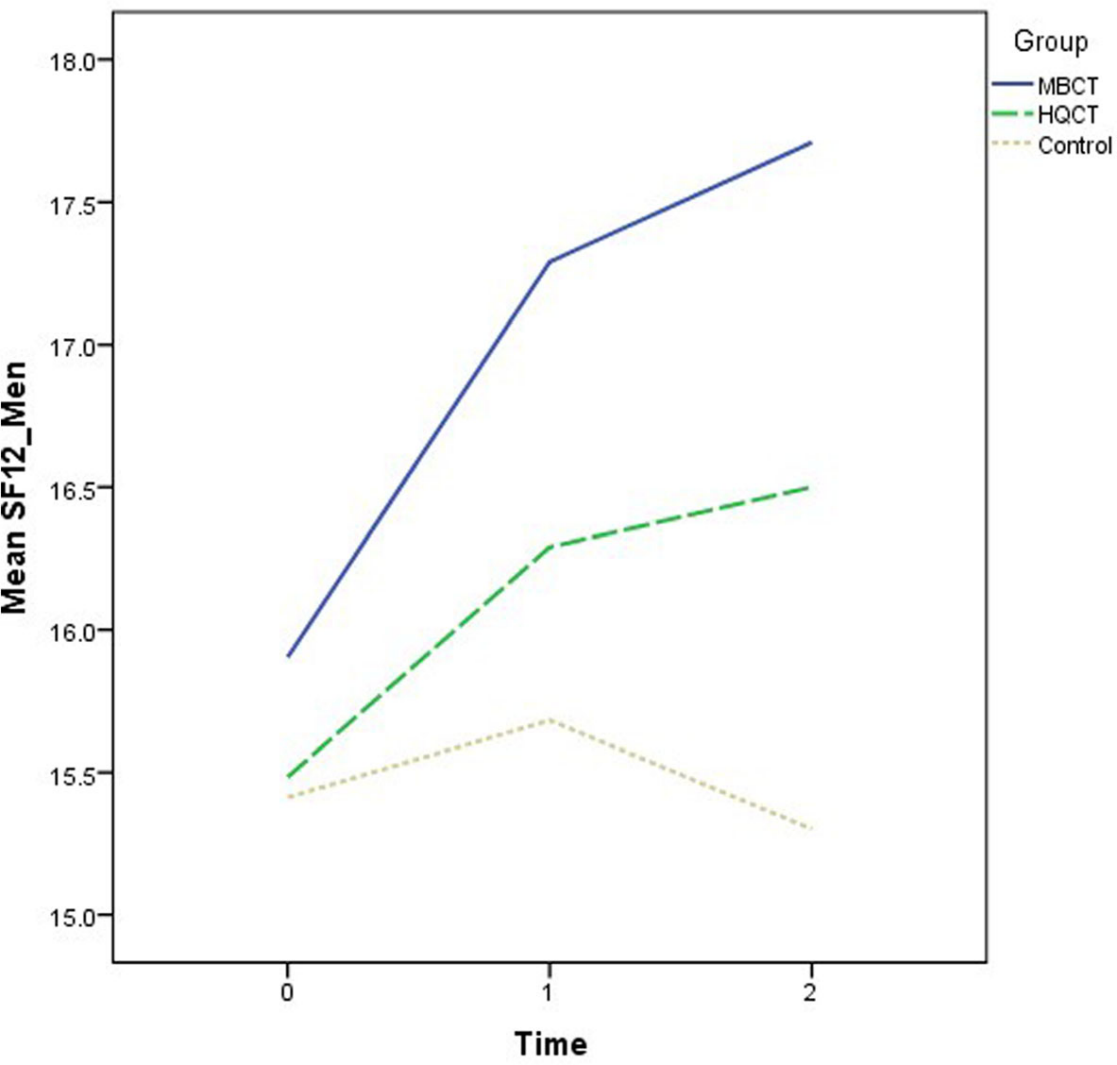
Growth trajectories of health status (Mental) for the three groups across three time points

**Fig. 5:**
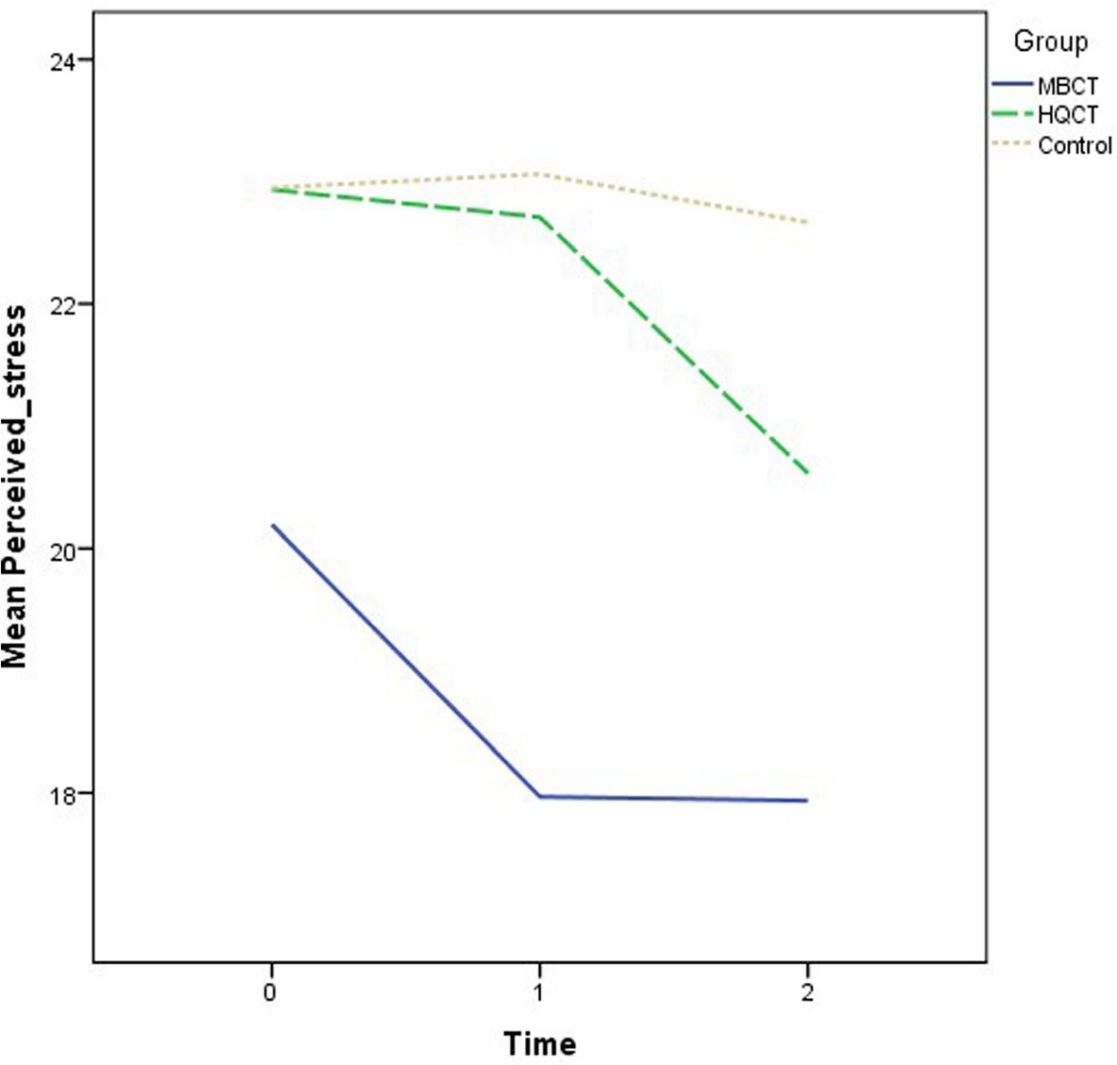
Growth trajectories of perceived stress for the three groups across three time points

**Fig. 6:**
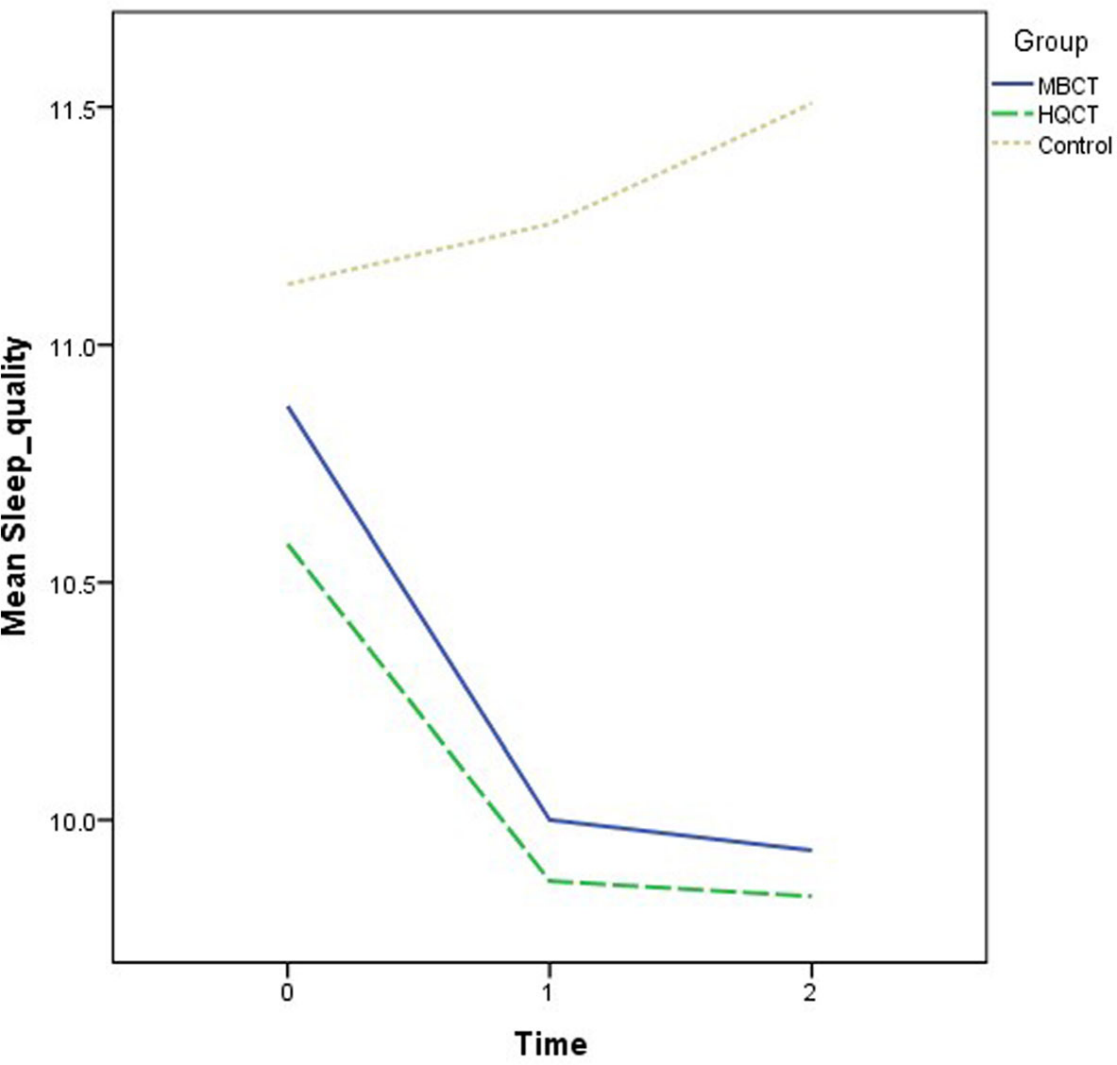
Growth trajectories of sleep quality for the three groups across three time points

**Fig. 7:**
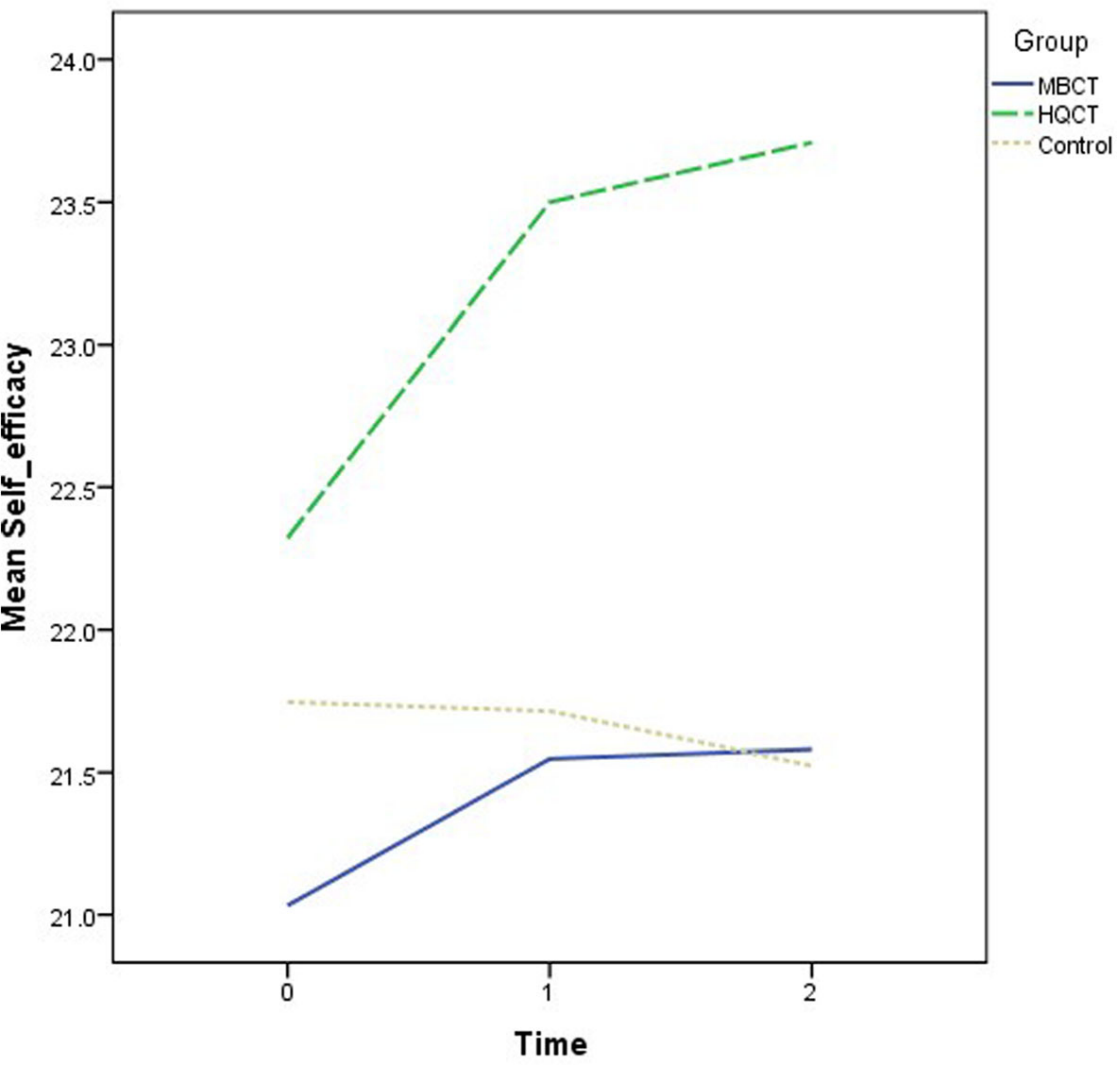
Growth trajectories of self-efficacy for the three groups across three time points

Recruitment and follow-ups took place over 12 months from November 2018 to November 2019. The demographic characteristics of the participants are summarized by group in Table 1. The mean age was 50.5 years (standard deviation = 10.9). The majority of the participants were female (70.6%) and had a secondary education level (55.1%). The descriptive statistics of the outcome variables at the three time points by group are also shown in Table 1. There were no significant differences in the demographic data and outcome variables at baseline when comparing groups. Various ANOVA results revealed insignificant effects on group: DASS-21 (F = 2.53, *p* = .09); SF12 (Physical) (F = .67, *p* = .52); SF12 (Mental) (F = 1.74, *p* = .18); CPSS (F = 2.44, p = .09); PSQI (F = 2.06, *p* = .13); and GSE (F = 1.18, *p* = .31). The growth trajectories of different outcome variables among three groups are depicted in Figs. 2, 3, 4, 5, 6 and 7.

**Table 1:** Demographic characteristics and clinical outcome variables of randomized participants

	MBCT (*n* = 62)	HQCT (*n* = 62)	WC (*n* = 63)			
	%	%	%			
Gender						
Male	29.0	32.3	27.0			
Female	71.0	67.7	73.0			
Education						
Primary	17.7	14.5	17.5			
Secondary	58.1	48.4	58.7			
Tertiary	24.2	37.1	23.8			
				MBCT vs. WC	HQCT vs. WC	MBCT vs. HQCT
	*Χ (SD)*	*Χ (SD)*	*Χ (SD)*	*ES*	*ES*	*ES*
Age	51.6 (9.5)	50.7 (10.6)	49.2 (12.4)			
DASS-21 (T0)	33.4 (12.2)	34.8 (12.7)	33.1 (14.3)			
DASS-21 (T1)	28.8 (11.7)	27.2 (11.6)	32.1 (14.2)	−0.27^a^	−0.49^a^	0.24^a^
DASS-21 (T2)	27.2 (11.4)	26.2 (10.8)	31.3 (13.0)	−0.33^b^	−0.5^b^	0.19^b^
SF12_Phy (T0)	12.6 (2.4)	12.6 (2.6)	12.7 (2.7)			
SF12_Phy (T1)	12.9 (2.5)	13.2 (2.7)	12.4 (2.6)	0.23^a^	0.34^a^	−0.12^a^
SF12_Phy (T2)	12.9 (2.4)	13.9 (2.5)	12.3 (2.6)	0.27^b^	0.64^b^	−0.40^b^
SF12_Men (T0)	15.9 (3.5)	15.5 (3.5)	15.4 (3.2)			
SF12_Men (T1)	17.3 (3.5)	16.3 (3.3)	15.7 (3.2)	0.33^a^	0.15^a^	0.17^a^
SF12_Men (T2)	17.7 (3.5)	16.5 (3.2)	15.3 (3.1)	0.56^b^	0.33^b^	0.23^b^
CPSS (T0)	20.2 (6.4)	22.9 (6.4)	23.0 (5.6)			
CPSS (T1)	18.0 (6.2)	22.7 (6.5)	23.1 (5.3)	−0.38^a^	−0.05^a^	0.21^a^
CPSS (T2)	17.9 (6.6)	20.6 (6.2)	22.7 (5.5)	−0.33^b^	−0.33^b^	0.00^b^
PSQI (T0)	10.9 (4.1)	10.6 (3.8)	11.1 (4.6)			
PSQI (T1)	10.0 (3.4)	9.8 (3.3)	11.3 (5.0)	−0.25^a^	−0.24^a^	−0.03^a^
PSQI (T2)	9.9 (3.5)	9.8 (3.5)	11.5 (4.7)	−0.32^b^	−0.28^b^	− 0.05^b^
GSE (T0)	21.0 (6.0)	22.3 (6.1)	21.8 (6.5)			
GSE (T1)	21.6 (6.4)	23.5 (5.4)	21.7 (5.8)	0.11^a^	0.21^a^	−0.10^a^
GSE (T2)	21.6 (6.3)	23.7 (5.9)	21.5 (6.5)	0.14^b^	0.27^b^	−0.13^b^

All comparisons of demographic data (between-group ANOVA or X^2^ tests) are nonsignificant, *p* > .05; ^a^Comparison between T0 and T1; ^b^Comparison between T0 and T2

*MBCT* Mindfulness-based cognitive therapy, *HQCT* Health qigong based cognitive therapy, *WC* Waitlist control, *DASS-21* 21-item Depression, Anxiety and Stress Scale, *SF12* Short-form 12, *Phy* Physical health status, *Men* Mental health status, *CPSS* Chinese Perceived Stress Scale, *PSQI* Pittsburgh Sleep Quality Index, *GSE* General Self-efficacy Scale

### Unconditional mean model

The results of the null model suggested that the development of a multilevel model is warranted because intercepts varied significantly across individuals (Wald *Z* = 8.778, *p* < .001), and the intraclass correlation suggested that about 77.3% of the total variability in DASS-21 lies between individuals. Thus, we could further develop multilevel models to explain the variability in intercepts within and between individuals.

### Linear and quadratic growth model

The individual variations of the growth rates of different outcome measures in respective groups are presented in Table 2. The results of the parameter estimates of fixed effects of Group × time interaction are presented in Table 3.

**Table 2:** Individual variation of the growth rates of different Y_it_ in different groups

	Initial status (*β*_00_)		Linear change (*β*_10_)		Quadratic change (*β*_20_)	
	Estimate	*t*	Estimate	*t*	Estimate	*t*
DASS-21						
MBCT	33.4	21.8***	-6.19	-4.03***	1.52	2.12*
HQCT	34.8	21.6***	-10.9	-7.45***	3.31	4.96***
WC	33.1	17.8***	-1.11	-1.10	.111	.244
SF12_Phy						
MBCT	12.6	40.7***	.427	.844	-.153	-.641
HQCT	12.6	35.9***	.629	1.17	.000	.000
WC	12.7	41.0***	-.492	-.780	.159	.528
SF12_Men						
MBCT	15.9	35.9***	1.87	3.62***	-.484	-2.02*
HQCT	15.5	35.1***	1.10	1.63	-.298	-.930
WC	15.4	39.0***	.595	1.11	-.325	-1.28
CPSS						
MBCT	20.2	24.9***	-3.32	-3.68***	1.10	2.56*
HQCT	22.9	27.8***	.710	.921	-.935	-2.62*
WC	23.0	32.0***	.365	.536	-.254	-.870
PSQI						
MBCT	10.9	21.5***	-1.27	-2.50*	.403	1.69
HQCT	10.6	23.0***	-1.05	-1.82	.339	1.24
WC	11.1	19.0***	.063	.102	.063	.222
GSE						
MBCT	21.0	27.0***	.758	1.11	-.242	-.768
HQCT	22.3	30.1***	1.66	1.64	-.484	-1.03
WC	21.7	27.4***	.048	.063	-.079	-.225

*MBCT* Mindfulness-based cognitive therapy, *HQCT* Health qigong based cognitive therapy, *WC* Waitlist control, *DASS-21* 21-item Depression, Anxiety and Stress Scale, *SF12* Short-form 12, *Phy* Physical health status, *Men* Mental health status, *CPSS* Chinese Perceived Stress Scale, *PSQI* Pittsburgh Sleep Quality Index, *GSE* General Self-efficacy Scale

*<.05; ***<.001

**Table 3:** Parameter estimates of fixed effects of group × time interaction effect for different Y_it_ between MBCT vs. WC, HQCT vs. WC, and MBCT vs. HQCT

	Initial status (*β*_01_)		Linear change (*β*_11_)		Quadratic change (*β*_21_)	
	Estimate	*t*	Estimate	*t*	Estimate	*t*
DASS-21						
MBCT vs. WC	−.170	−.143	2.54	2.89***	−.707	−1.67
HQCT vs. WC	−1.71	−.710	3.84	5.20***	− 3.20	−3.97***
MBCT vs. HQCT	.683	.311	−.876	−1.10	1.78	1.82
SF12_Phy						
MBCT vs. WC	.027	.118	−.460	−1.03	.156	.811
HQCT vs. WC	.009	.020	−.786	−3.14**	.159	.402
MBCT vs. HQCT	−.041	−.090	.504	2.12*	.153	.438
SF12_Men						
MBCT vs. WC	−.285	−.983	−.459	−3.73***	.079	.454
HQCT vs. WC	−.060	−.104	−.568	−2.10*	−.027	−.066
MBCT vs. HQCT	−.481	−.788	−.375	−1.31	.185	.463
CPSS						
MBCT vs. WC	1.56	2.93**	.520	2.28*	−.675	−2.61**
HQCT vs. WC	.017	.016	−.345	−.336	.682	1.48
MBCT vs. HQCT	3.26	2.86**	−.030	−.073	−2.03	−3.65***
PSQI						
MBCT vs. WC	.224	.589	.281	1.99*	−.170	−.910
HQCT vs. WC	.690	.936	.490	1.72	−.275	−.695
MBCT vs. HQCT	−.240	−.378	.079	.337	−.065	−.181
GSE						
MBCT vs. WC	.331	.601	−.196	−1.04	.081	.344
HQCT vs. WC	−.844	−.802	−.753	− 1.66	.405	.691
MBCT vs. HQCT	1.39	1.31	.390	.855	−.242	−.428

*Note*. * < .05; ** < .01; *** < .001; *MBCT* Mindfulness-based cognitive therapy, *HQCT* Health qigong based cognitive therapy, *WC* Waitlist control, *DASS-21* 21-item Depression, Anxiety and Stress Scale, *SF12* Short-form 12, *Phy* Physical health status, *Men* Mental health status, *CPSS* Chinese Perceived Stress Scale, *PSQI* Pittsburgh Sleep Quality Index, *GSE* General Self-efficacy Scale

Regarding the intervention effect of MBCT compared with WC, participants in the former group showed significant time effect and time × group interaction effects on DASS-21, SF12 (Mental), CPSS, and PSQI. In terms of the primary outcome, individuals in the MBCT group revealed significant decrease (*β* = − 6.19, *t* = − 4.03, *p* < .001) of mood disturbance and significant increase in the rate of change (*β* = 1.52, *t* = 2.12, *p* < .05). With regard to secondary outcomes, participants in the MBCT group indicated significant improvement of mental health status (*β* = 1.87, *t* = 3.62, *p* < .001), sleep quality (*β* = − 1.27, *t* = − 2.50, *p* < .05), and perceived stress level (*β* = − 3.32, *t* = − 3.68, *p* < .001). Effect size statistics also indicated that the MBCT group had small to moderate effects (*d* = 0.25 to 0.56) on the above outcome variables at post-treatment and follow-up assessments compared with the WC group.

Compared with the WC group, participants in the HQCT group showed significant time and time × group interaction effects on DASS-21. Individuals in the HQCT group demonstrated significant decrease (*β* = − 10.9, *t* = − 7.45, *p* < .001) of mood disturbance and significant increase in the rate of change (*β* = 3.31, *t* = 4.96, *p* < .001). With regard to secondary outcomes, significant time × group interaction effects on both SF12 (Physical) and SF12 (Mental) were also noted. Effect size statistics also indicated that the HQCT group had small to moderate effects (*d* = 0.15 to 0.64) on the above outcome variables at post-treatment and follow-up assessments compared with the WC group.

Comparable intervention effects between MBCT and HQCT were noted across different outcome variables. In terms of primary outcome, both interventions led to significant reductions of mood symptoms, with relatively more reductions in the HQCT group during post-treatment (*d* = 0.24) and follow-up (*d* = 0.19) assessments. With regard to secondary outcomes, participants in the MBCT group showed much improvement in mental health status (*d* = 0.17 to 0.23) as compared with counterparts in the HQCT group. On the contrary, the latter showed much enhancement in physical health status (*d* = 0.12 to 0.40) across time.

### Individual growth models with time-invariant and time-varying predictors

Table 4 shows the parameter estimates of the individual growth model of the MBCT group and HQCT group on DASS-21. Specifically, time-invariant predictors (gender, age, education) were nonsignificant in predicting linear or quadratic change of DASS-21 for both groups. For the MBCT group, SF12 (Mental) and CPSS were significant time-varying predictors of DASS-21. Particularly, participants in the MBCT group with increasing mental health status and decreasing perceived stress level tended to report an attenuation of mood symptoms over time. For the HQCT group, SF12 (Physical), CPSS, and GSE were significant time-varying predictors of DASS-21. More specifically, participants in the HQCT group with increasing physical health status, enhancing self-efficacy level, and lowering perceived stress level tended to report a reduction of mood symptoms over time.

**Table 4:** Parameter estimates of fixed effects of the time-invariant and time-varying predictors within the individual growth model of the MBCT group and HQCT group on DASS-21

	MBCT				HQCT			
	Estimate	Standard error	*t*	*p*	Estimate	Standard error	*t*	*p*
Initial status (*π*_*oi*_)								
Constant (*β*_00_)	60.0	13.4	4.46	.000	63.1	12.7	4.98	.000
Gender (*β*_01_)	−3.59	2.98	−1.21	.233	−2.99	2.80	−1.07	.290
Age (*β*_02_)	−.202	.150	−1.36	.180	−.274	.147	−1.86	.068
Education	−1.12	2.24	−.500	.619	−2.50	2.10	−1.19	.238
Linear change (*π*_1*i*_)								
Constant (*β*_10_)	−15.0	13.1	−1.15	.254	−29.2	14.3	−2.03	.047
Gender (*β*_11_)	2.73	3.56	.768	.446	7.06	3.92	1.80	.077
Age (*β*_12_)	.033	.180	.182	.857	.259	.205	1.27	.211
Education	1.28	2.70	.473	.638	5.14	2.93	1.76	.085
Quadratic change (*π*_2*i*_)								
Constant (*β*_20_)	3.51	5.98	.587	.560	13.27	6.24	2.13	.038
Gender (*β*_21_)	−.947	1.62	−.586	.561	−2.82	1.71	−1.65	.104
Age (*β*_22_)	.023	.082	.281	.779	−.137	.089	−1.54	.129
Education	−.516	1.24	−.416	.679	−2.33	1.27	−1.83	.072
SF12_Phy (*β*_30_)	−.289	.261	−1.11	.271	−.594	.270	−2.20	.029
SF12_Men (*β*_40_)	−.779	.213	−3.66	.000	−.431	.237	−1.82	.071
CPSS (*β*_50_)	.307	.143	2.14	.034	.514	.138	3.74	.000
PSQI (*β*_60_)	.134	.214	.625	.533	.069	.222	.312	.756
GSE (*β*_70_)	−.180	.122	−1.48	.141	−.297	.144	−2.06	.041

*Note*. *MBCT* Mindfulness-based cognitive therapy, *HQCT* Health qigong based cognitive therapy, *DASS-21* 21-item Depression, Anxiety and Stress Scale, *SF12* Short-form 12, *Phy* Physical health status, *Men* Mental health status, *CPSS* Chinese Perceived Stress Scale, *PSQI* Pittsburgh Sleep Quality Index, *GSE* General Self-efficacy Scale

## Discussion

This study compared the intervention effects of MBCT and HQCT with the WC group in treating depression and anxiety within a Chinese context. Our first hypothesis was supported. Significant individual reductions of mood symptoms after completion in both the MBCT and HQCT groups were indicated. Our findings were consistent with previous research on the beneficial impacts of MM and HQ on depression and anxiety [14, 20]. Previous research only showed small-to-moderate efficacy for reducing mood symptoms [46] or without reliably effects being shown at follow-up assessments [13]. However, findings of the present study indicated a sustained moderate effect size during follow-up for both MBCT and HQCT. The enhanced effect of integrating CBT into different forms of MBI is implied.

Apart from the mood symptoms, both intervention groups also demonstrated significant improvements on health statues as compared with the WC group. More specifically, MBCT is more conducive to improving mental health status whereas HQCT is more beneficial to physical health outcomes. Our second hypothesis was confirmed. According to the individual growth models, enhanced mental health status lead to attenuation of mood symptoms in the MBCT group. As such, the essence of mindful awareness and cognitive restructuring of thoughts in MBCT should be the major ingredients in making changes in mental health outcomes [47]. Thus, consistent with previous research findings [48, 49], results in this study also indicated the significant impact of MBCT on mental health, which is an imperative component in changing mood symptoms. On the other hand, the role of physical health in HQCT was highlighted, as evidenced by the individual growth model. Previous research studies usually revealed the influence of mood symptoms on physical health functioning [50, 51]. Instead, findings of the present study put physical health in an alternative perspective in which it was viewed as an imperative predictor of mood changes. Consistent with previous findings [52, 53], HQ per se, as well as the combined effect with CBT, can produce significant benefits for physical health which ultimately leads to a promising effect on mood symptoms.

In addition, participants in the HQCT group showed slightly greater reduction of mood symptoms than those in the MBCT group, albeit with small effect size being indicated. Our third hypothesis was partially verified. It appears that more benefits can be derived from movement-based MBI intervention as compared with mind-based MBI for this population. The predominant emphasis on physical health in HQCT may shed light on its applicability on people with a Chinese cultural background. As HQ is always perceived as mind-body exercises or meditative movement [26, 54], people from a Chinese cultural background consider such exercise as a health-preserving activity which is a crucial indicator of health status [55]. Moreover, compared with conventional exercise, HQ is a form of exercise that uses movement in conjunction with moving vital energy (or qi) throughout the entire body. Thus, it should lead to additional neurophysiological or biological effects on mood symptoms [17].

Moreover, the somatization tendency [56] as espoused in Chinese culture may also explain the greater effect of HQCT on mood change. Essentially, there is strong “holism” ideation in Chinese culture. A person is usually conceived “holistically” as a psychosomatic process [57], thus there is no dichotomy of mind versus physical body as both are perceived as the same substance [58]. Therefore, such holistic conception may lead people to link physical and mental health easily under the influence of Chinese culture. This could be explained by the significant effects on both physical health and mental health statuses after participating in HQCT. Therefore, movement-oriented meditation like HQCT should be much more effective than the static form like MBCT in improving depression and anxiety [59].

Attenuation in perceived stress is the common predictor of mood changes in both MBCT and HQCT, which can further support the stress reduction effect of both interventions. Perceived stress reactivity could operate in specific pathways toward the development of anxious or depressive symptoms [60, 61]. Thus, echoing with previous findings [19, 62], it is no wonder perceived stress is one of the core elements causing the mood changes in MBCT and HQCT. Self-efficacy is another predictor of mood changes in HQCT but not in MBCT. Perhaps within HQCT, people gained a sense of accomplishment by learning a new skill set which is perceived as less physically or cognitively demanding [34]. Thus, enhancement of self-efficacy over time due to performance accomplishments [63] can eventually lead to mood symptom improvement [64]. On the other hand, significant sleep improvement was only observed in MBCT but not in HQCT. Despite similar trajectory was observed between two interventions (Fig. 6), only MBCT showed statistically significant improvement on sleep parameter. Perhaps merely the core principles of mindfulness, involving experiential awareness, attentional control, and acceptance, can directly target different vulnerabilities associated with sleep disturbances [65].

## Limitations and conclusions

First, despite the fact that a comparison of MBCT and HQCT in this study can provide useful information for appraising the effects of these two integrated interventions, the lack of a CBT-only control group may undermine the conclusion to be made regarding the additional effect of two forms of MBIs on top of CBT. Second, there was no breakdown of the mood disorders into different subtypes in this study. Considering MM or HQ can have distinct mechanisms of action on the effects of depression versus anxiety [27, 66], it is suggested that specific interpretations can be made regarding different modes of intervention as applied to depression and anxiety, respectively. Besides, meta-analysis suggested that although there are positive signs of the value of transdiagnostic CBT for anxiety and depression, there is as yet insufficient evidence to recommend its use in place of disorder-specific CBT [67]. Thus, comparison of transdiagnostic-based versus disorder-specific HQCT and MBCT is worthy of further investigation. Third, only a small sample was recruited in this study, consisting of a group of motivated participants from a single clinic, so they cannot be representative of a wider spectrum of individuals with mood disorders. Lastly, regarding the elements involved in the intervention, Baduanjin was chosen in this study as the HQ practice in HQCT. However, there are different forms of HQ practice at present, such as “The Five-Animal Play” or Tai Chi Chuan [17], with lots of research having been done regarding the latter’s effects on depression and anxiety [68]. Thus, further research could be considered to compare different forms of HQ practice. Despite the aforesaid limitations, this study can make a contribution by proving the beneficial effects of HQCT and MBCT over WC in bringing about mood changes. More specifically, the physical health emphasis of HQCT can even make it more acceptable and effective in a Chinese population.

## Supplementary Information

**Additional file 1.**

## Data Availability

Available upon request.

## Abbreviations

CBT: Cognitive Behavioral Therapy
MBI: Mindfulness-based intervention
MM: Mindfulness meditation
MBCT: Mindfulness-based cognitive therapy
HQ: Health qigong
HQCT: Health qigong-based cognitive therapy
RCT: Randomized controlled trial
WC: Waitlist control
DASS: Depression Anxiety and Stress Scale
SF-12: Short-form-12
CPSS: Chinese Perceived Stress Scale
PSQI: Pittsburgh Sleep Quality Index
CGSS: Chinese General Self-efficacy Scale
LMM: Linear mixed model
IGC: Individual growth curve
AIC: Akaike information criterion
